# Development of an Exergame to Deliver a Sustained Dose of High-Intensity Training: Formative Pilot Randomized Trial

**DOI:** 10.2196/games.7758

**Published:** 2018-03-27

**Authors:** Thomas McBain, Matthew Weston, Paul Crawshaw, Catherine Haighton, Iain Spears

**Affiliations:** ^1^ Academy of Sport and Physical Activity Sheffield Hallam University Sheffield United Kingdom; ^2^ School of Social Sciences, Humanities & Law Teesside University Middlesbrough United Kingdom; ^3^ Department of Social Work, Education & Community Wellbeing Northumbria University Newcastle upon Tyne United Kingdom; ^4^ Institute of Health & Society Newcastle University Newcastle upon Tyne United Kingdom; ^5^ Pro-Football Support Ltd Huddersfield United Kingdom

**Keywords:** high-intensity interval training, video games, heart rate, boxing, metabolic syndrome

## Abstract

**Background:**

Sport science can play a critical role in reducing health inequalities. The inverse relationship between life expectancy, cardiorespiratory fitness, and socioeconomic status could be addressed by performing high-intensity training (HIT), delivered in a class salient and accessible approach. Commercially available exergames have shown encouraging compliance rates but are primarily designed for entertainment purposes rather than focusing on health-related outcomes. A serious game tailored toward delivering an exercise stimulus, while reducing the aversive protocols associated with HIT, could be beneficial to engage and improve health outcomes in socially deprived males.

**Objective:**

The aims of this study were to develop an exergame capable of delivering HIT and evaluate the effect on selected health outcomes in men recruited in regions of socioeconomic deprivation.

**Methods:**

We conducted an exploratory trial in our target population, and participants were allocated to intervention (n=14) or control groups (n=10) by third-party minimization. The intervention was a 6-week training program consisting of three sessions of exergaming per week. The sessions involved a structured warm-up, then brief intermittent repetitions in the form of boxing rounds (10 s, 20 s, and 30 s) against their peers with a work/rest ratio of 0.25.

**Results:**

Retention to the intervention was 87.5% (21/24). Over the duration of the intervention, session attendance was 67.5% (170/252); repetition mean and peak heart rates (% of maximal) and session ratings of perceived exertion (AU, arbitrary units) were 86.3 (5.4%), 89.9 (6.1%), and 7.5 (2.2 AU), respectively. The effect of the intervention, when compared with the control, was a likely small beneficial improvement in predicted maximum oxygen consumption (VO_2_ max, 3.0; 90% confidence limits ±2.6%). Effects on body mass, waist circumference, and blood pressure were either trivial or unclear.

**Conclusions:**

Over the 6-week intervention, the exergame delivered a consistent and sustained dose of HIT, with some beneficial effects on aerobic fitness in the target population.

**Trial Registration:**

ClinicalTrials.gov NCT03477773; https://clinicaltrials.gov/ct2/show/NCT03477773 (Archived by WebCite at http://www.webcitation.org/6yDLgVs35)

## Introduction

The latest government audit of university-based research in the United Kingdom has highlighted the need for more sport and exercise research to target health inequalities that persist in society [1]. There are strong inverse relationships between life expectancy, cardiorespiratory fitness, and socioeconomic status [2-4]. As these trends are exacerbated in males, improving fitness in men living in regions of socioeconomic deprivation could be important for addressing health inequalities. High-intensity training (HIT) is a time-efficient way to improve fitness over a short duration [5], and a growing body of evidence advocates this form of exercise for public health interventions [6]. HIT is known to stimulate a combination of central and peripheral adaptations promoting an enhanced availability, extraction, and utilization of oxygen [7,8]. Although these findings are encouraging, these previous studies have tended to prescribe exercise with a focus on cycling and running [9-12]. Some argue that the psychologically aversive nature of high-intensity exercise means that this training will not be adopted or maintained by many people [13]. Thus, although the physiological benefits of HIT are unequivocal, there is ongoing debate about its relevance across populations, particularly for those less-motivated individuals. Finding ways to improve its acceptability could be the key to improve the wider acceptance of HIT.

The concept of exergaming, or active video gaming, has been around since the 1980s and affords unrivalled opportunities to reward exercise through visual, audio, haptic, and mental stimuli. Exergaming-based interventions have understandably shown improved rates of compliance, adherence, and social inclusion (eg, see [2]). Despite this, however, and after nearly a decade of research, the potential for exergaming to improve levels of fitness on a population level has not been realized [14]. A possible reason is that most of the research is based on commercially available exergames that are designed primarily for entertainment. In other areas of health research, such as physiotherapy, researchers have overcome these shortfalls by creating their own exergames specifically geared to the physiological needs of the clinical or at-risk population. These serious games are purposively designed to train physiological systems at a level that is sufficient to cause positive adaptation. Importantly, these serious exergames have succeeded in improving health-related outcomes (eg, balance and falls risk) and demonstrate their potential for use in other health professions [15].

A major challenge with improving HIT and exergaming is to translate positive laboratory-based findings into interventions that can directly affect those individuals who need it most and can also be administered on a larger scale [16,17]. Whether or not the concept of serious exergaming can contribute in this regard requires, first, that a game is tailored toward reducing the aversive protocols associated with HIT and second, that the intervention is capable of improving fitness in our target population. Given that the commercially available games are known to deliver, at best, low to moderate levels of exercise [18], our first aim was to describe the development of a serious exergame for this purpose. Our second aim was to quantify its real-world fidelity and potential to improve fitness and health outcomes in males recruited in regions of socioeconomic deprivation over a 6-week intervention.

## Methods

### Development of the Game and Intervention

**Figure 1:**
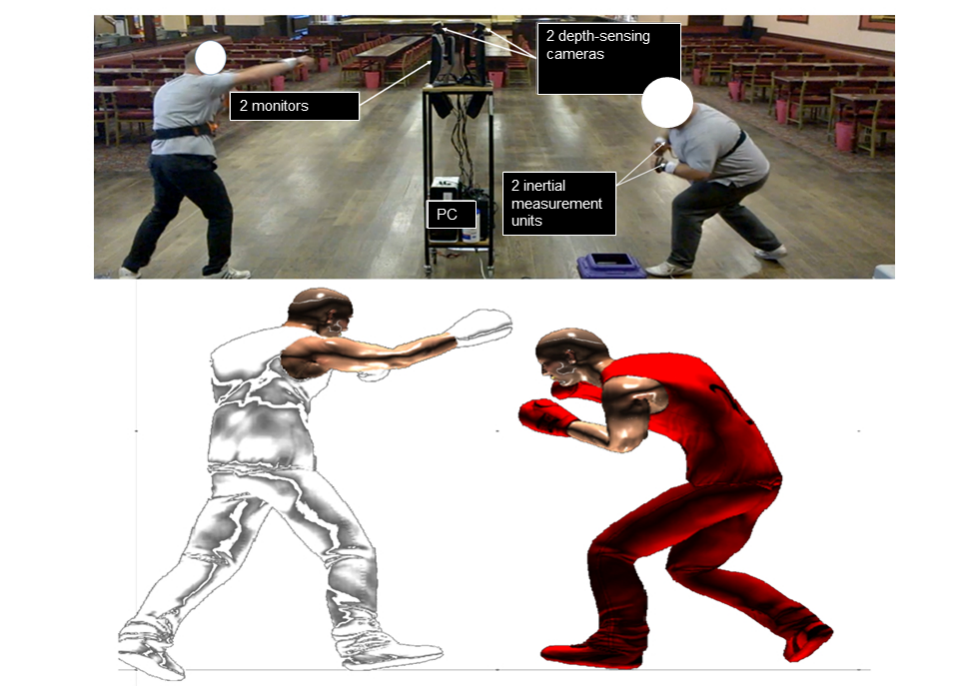
The exergaming system comprising hardware and software with real-time avatar mapping.

**Figure 2:**
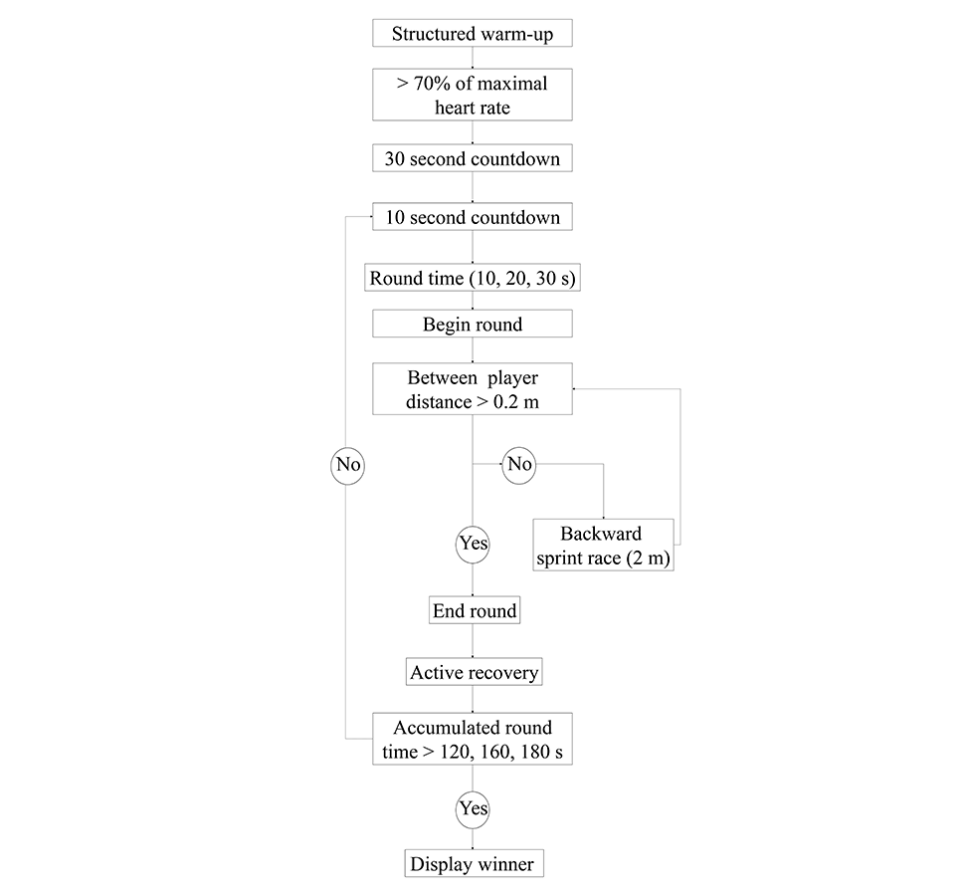
Machine state variables used in controlling the game.

During the preintervention period, we held regular focus groups comprising in-house participants, computer programmers, and exercise scientists with specialisms in delivering HIT. Together, we explored opportunities to gamify HIT protocols. Boxing was an obvious theme for gamification as it involves high-intensity exercise [19], and the subculture already exists within our target population of lower working-class males of the North-East of England [20,21]. The complete set of hardware used in the trials comprised a single computer with a dedicated graphics card (GeForce GTX 960, [22]), two standard 23-inch monitors, two Kinect for Windows sensors, four wrist-mounted wireless inertial measurement units (developed in-house to reduce latency in the Kinect datastream), and two wireless chest-worn heart-rate monitors (Polar RS400, Polar Electro Oy, Kempele, Finland). In addition, the participants wore boxing-specific resistance bands (ShadowBoxer Pty, Australia) to increase levels of exertion and reduce eccentric muscle contractions during the deceleration phase of punching. The movement data from the sensors were streamed to our gaming engine [23] via a User Datagram Protocol with a latency of approximately 15 ms. Input from the skeletal tracking module of the Kinect system and inertial measurement units were converted to a digital avatar using purpose-written functions to convert each body segment long-axis (19 in total) into three-dimensional quaternions, as can be seen with the hardware setup in Figure 1. The C# programming language was used throughout to create scripts for gameplay (Figure 2).

Commercial games are primarily designed for entertainment and recreation for younger populations and tend to have colorful and visually busy game and user interfaces [24]. Furthermore, commercially available games are mostly designed for enjoyment and not based on basic exercise principles. For games to promote beneficial changes in health, they need to engage physiological systems in a meaningful manner that is relevant for the function being trained [25]. Our philosophy was to ensure that the gameplay was simple and, where possible, true to the rules of boxing. Each avatar was assigned collision objects with inertial characteristics on the trunk, head, and hands, thus enabling the physics engine to superimpose realistic joint movements in response to being hit. As in real boxing, the primary mechanism for scoring was to successfully land a punch on either the trunk or head of the opponent. The number of points awarded per punch was the product of the current heart rate (expressed as a percentage of maximal) and speed of impact (ms^-1^).

**Figure 3:**
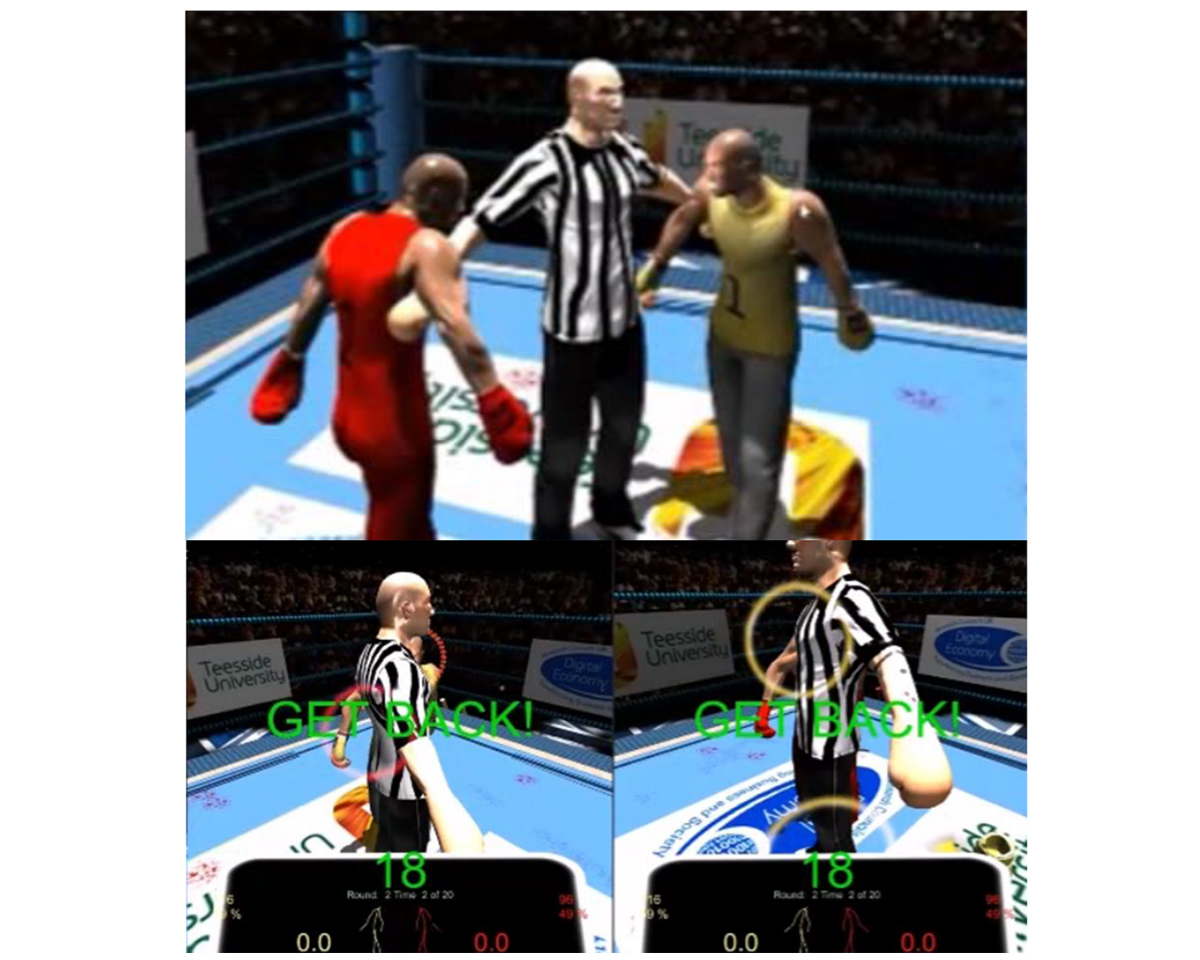
The three visual outputs communicating instructions and actual game play to the users.

Thus, for example, a punch landing to the head with a relative speed of 9 ms^-1^and at 95% of maximal heart rate (ie, 95% HRmax) was awarded 8.55 points (ie, 9 x 0.95). Each participant was provided with biofeedback in the form of a partial arc positioned around the opponent’s head; the angle subtended by this arc was linked to the current heart rate (Figure 3).

For example, when player 1 was working at 50% of maximal heart rate, an arc of 180^o^ was displayed around the head of player 2. The players were made aware that the angle of the arc was proportional to their current training intensity, which in turn contributed strongly to punch power and points scoring. They were also aware that large global displacements of their center of mass (eg, bouncing up and down) would increase this intensity but with a lag of approximately 20 to 30s [26]. This arc was visible in their monitor only and was active throughout the exergaming session (ie, during both repetitions and rest), essentially allowing the participants to self-regulate training intensity per the oncoming requirements of the game. Although we kept on-screen text to a minimum, we elected to display a countdown timer during some periods. These were during the final 30 s of active recovery to encourage the elevation of training intensity in preparation for a repetition and during the latter 10 s of each repetition to encourage all-out punching right up until the bell. Following preliminary tests, we removed the ability to block punches as this was observed to be energetically undemanding; thus, the only forms of defense were to attack before being hit or to dodge the oncoming punch using whole body movements. For similar reasons, we limited the periods of allowable close-up punching. Specifically, after 1 s of close-up punching, the referee interrupted the fight (shown in the striped shirt in Figure 3) and ordered both participants to race back to their respective corners (ie, 3 m backwards of backwards stepping). The winner of each mini race received an additional 50 points; equivalent to approximately 6 to 7 well-timed punches. After several iterations of observing kinematics, heart rates, and tactics during gameplay, we prepared the exergaming system (ie, both hardware and software) for use in the target settings.

### Participants

**Figure 4:**
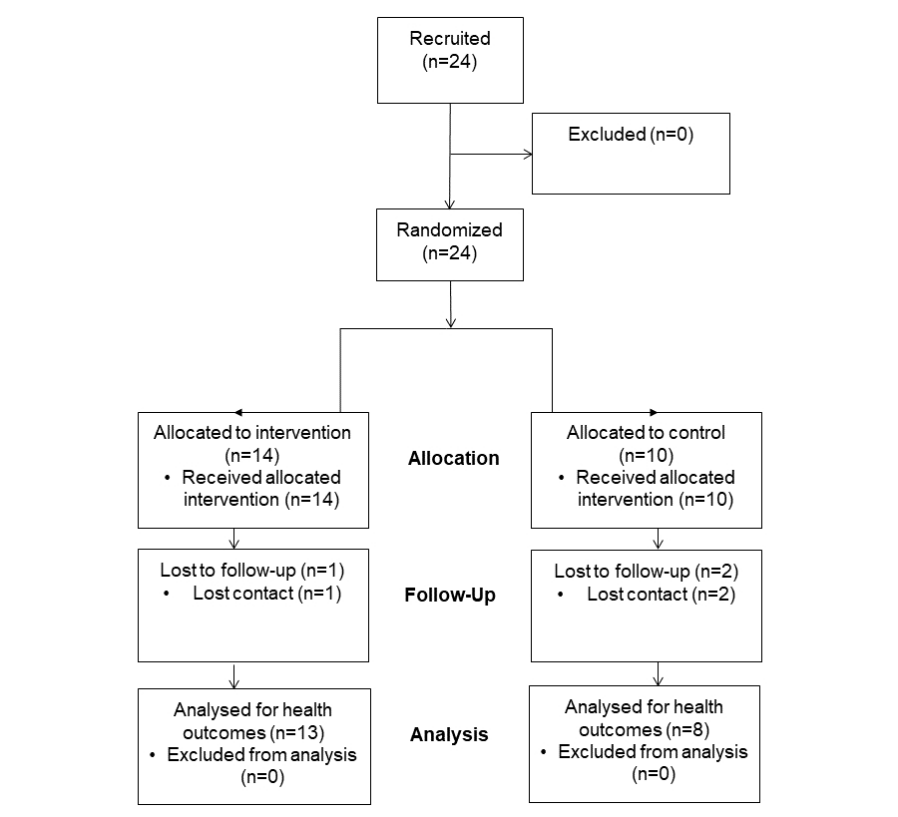
Participant flow though the trial.

We conducted a 6-week exploratory controlled trial designed to assess the fidelity of the game in terms of delivering the intended training stimulus and to examine the effect of the intervention on selected health outcomes. As appropriate for an exploratory trial, we did not conduct formal sample size estimation a priori, rather the CIs would be used to inform future trials. Men are commonly referred to as a hard-to-reach population to engage in health promotion activities [27], where a targeted recruitment approach at locations predominantly attended by men may facilitate uptake of participants. Therefore, to maximize recruitment within our intended population, relevant gatekeepers were approached at institutions positioned within regions of social deprivation. Thus, two settings used for recruitment and the trials were a social club and mosque, both situated within deprived regions of Middlesbrough, United Kingdom (TS1 and TS4). Recruitment in the social club relied heavily on the gatekeeper (club secretary), whereas uptake of South Asian men from a local mosque was more straightforward; all 9 mosque participants were recruited from a single demonstration (and we had similar experiences in previous iterations). Presumably, the intervention was culturally salient in this population; the North of England has developed some famous prominent Muslim boxers (eg, Amir Kahn and Naseem Hamed) in recent years, and when delivered in the safe form, was acceptable to the respected leaders of the religious groups (ie, the Imam of the mosque). A total of 24 males were recruited into the trial (Figure 4) using relevant gatekeepers at institutions positioned within regions of social deprivation. Two recruitment drives (October 2014 and February 2015) took place, and these involved live demonstrations of the technology followed by word-of-mouth and snowballing approaches. The exergaming system was important in this recruitment process because it provided something tangible and interesting to engage potential participants.

Inclusion criteria were deliberately broad to maximize recruitment, that is, apparently healthy, as defined by ACSM guidelines and in the age range of 18 to 55 years [28]. Participants were given a £15 shopping voucher upon completion of follow-up measures (week 7) irrespective of their adherence to the intervention protocol or group allocation. The intervention took place from November 2014 to December 2014 (social club) and March 2015 to April 2015 (mosque). Postcode data were analyzed to match against indices of multiple deprivation in lower-layer super output areas to check whether the cohort fell within our target population. Two participants (8%, 2/24) lived in the least deprived 20% (decile 8), 20 participants (83%, 20/24) lived in the most deprived 20% areas in England (decile 2), and 1 participant (6%, 1/24) lived in the most deprived 1% of areas (decile 1). The participants were broadly split between white (58%, 14/24) and South Asian (42%, 10/24). Body mass index [29] and waist circumference [30] cut-points were adjusted for ethnicity. Accordingly, 63% (15/24) had abdominal obesity, 37.5% (9/24) were overweight, and 42% (10/24) were obese. Within this group, the 45 to 50 years category was most common (33%, 8/24), followed by 18 to 24 years (29%, 7/24), 25 to 34 years (17%, 4/24), 35 to 44 years (17%, 4/24), and 51 to 55 years (4%, 1/24) categories.

A third-party minimization process using baseline measures of age, waist circumference, and predicted maximum oxygen consumption (VO_2_ max) was used to remove bias in group allocation. The control group was instructed to maintain their current physical activity levels and inform the researchers should any changes arise during the intervention period. One of the participants in the control group decided to embark on his own fitness program following the preintervention tests but was included in the data analysis. Participant flow through the trials is shown in Figure 4, whereby 3 participants were lost to follow-up (control=2, intervention=1). Overall retention to the intervention that encompassed baseline and follow-up measures was 87.5% (21/24).

To explore perceptions of the exergame and the HIT regime, semistructured interviews were conducted with 5 intervention participants following the 6-week training period, which were analyzed semantically. The study was approved by the ethics committee of Teesside University, United Kingdom, and written informed consent was obtained from all participants.

### Measures

Participants’ baseline characteristics (mean [SD]) are shown in Table 1. All measures were assessed pre and post intervention. Blood pressure was collected on the left arm positioned at heart height with the subjects in a seated position by an automatic upper arm blood pressure monitor (Omron MX13). Measures were made at least three times at 3-min intervals, where an average of the two lowest measures was used for analysis [31]. Waist circumference was measured using the World Health Organization guidelines [32]. Predicted VO_2_ max was obtained by performing a submaximal 8-min ramped step test [33]. Heart rate response (Polar T34; PolarElectro OY, Kempele, Finland) and simultaneous breath-by-breath expired gas were collected using a portable indirect calorimeter (Cosmed K4 b2; Rome, Italy), calibrated per the manufacturer’s guidelines. Individual HRmax was estimated [34] and plotted against VO_2_ data for the determination of predicted VO_2_ max.

### Intervention Delivery

Evidence recommends a minimum duration of 12 weeks for a HIT protocol to promote favorable changes in blood pressure and anthropometric measurements of obesity [35]. However, a 6-week intervention was selected, as a minimum of 13 sessions (0.16 work/rest ratio) is sufficient to elicit moderate improvements in VO_2_ max in sedentary individuals [5]. Additionally, there is still ambiguity regarding the optimal work-to-rest ratio when designing HIT interventions, particularly in populations with varied age, baseline fitness, and training experience [36]. Therefore, longer duration HIT models (1-4 min) were deemed unsuitable for our target population [37].

Furthermore, minigames (such as the current exergame) have short life spans, where adherence to a longer intervention (eg, 12 weeks) may diminish over time and influence health outcomes. This was evident from a 12-week pilot study (unpublished data) using an exergame in the same population that saw attendance drop from 53% during week 2 to 16% during week 12.

Participants allocated to the intervention group were invited to attend three sessions of exergaming per week. At the beginning of the exergaming session, participants were required to complete a 6-min structured warm-up consisting of a series of exercises on a 210 mm step until both participants reached >70% HRmax. Session workloads with volumetric progression were set automatically once the user’s identifying information was entered. The session workloads were 120s-, 150-s, and 180-s of work during weeks 1 and 2, weeks 3 and 4, and weeks 5 and 6, respectively.

To avoid staleness, the repetition lengths (10, 20, or 30-s) were randomly selected at the beginning of each round. We set the work-to-rest ratio at 1:4, and thus, the respective repetitions were followed by 40, 80, or 120-s of active recovery. Participants were instructed to perform the repetitions at an intensity ≥85% HRmax. Each exergaming session took approximately 30 to 40 min to complete, including equipment set-up, warm-up with additional enjoyment, and task immersion questionnaires upon completion of the HIT bouts (not reported here). Heart rate responses were taken within repetitions and therefore, did not include any of the recovery period. This, therefore, avoided an overestimation of physiological load, which can occur when heart rate continues to rise after exercise cessation [26].

### Statistical Analysis

Data are presented as mean (SD). Exergaming session attendance was calculated using descriptive statistics. Training data (heart rate [repetition mean and peak], rating of perceived exertion [RPE]) were analyzed using a mixed linear model with a random intercept (Statistical Package for the Social Sciences [SPSS] version 23 [IBM Corp]).

This approach enabled us to calculate (1) the within-participant variability in the training dose [38], expressed as an SD; (2) the effect of HIT repetition duration (entered as a fixed effect) on heart rate and RPE; and (3) the change in heart rate and RPE across the 6-week HIT intervention, with session number (1-18) entered as a fixed effect. A priori, we defined a minimal practically important difference (MPID) in training heart rates as two percentage points, given that when training at high-intensity, this difference influences the adaptive response [39].

The MPID for RPE was set at one arbitrary unit (AU) on the Borg CR10 Scale, representing a full increment change on the scale. Inferences were then based on the disposition of the 90% confidence limits (CLs) for the mean difference to these MPID; the probability (percent chances) that differences in heart rate and RPE between HIT repetitions of different durations or across the 6-week intervention were substantial (>2 percentage points, >1 AU) or trivial was calculated as per the magnitude-based inference approach described by Batterham and Hopkins [40], an approach that has been advocated within user research [41].

These percent chances were qualified via probabilistic terms assigned using the following scale: 25 to 75%, possibly; 75 to 95%, likely; 95 to 99.5%, very likely; and >99.5%, most likely [42]. To determine the magnitude of the within-participant variability in our training heart rate and RPE, the values were doubled and then interpreted against aforementioned MPID. All training data effects were evaluated mechanistically, whereby if the 90% CL overlapped the thresholds for the smallest worthwhile positive and negative effects, the effect was deemed unclear [42]. The effect of HIT on our outcome measures was determined using a custom-made spreadsheet [43], with the baseline value of the dependent variable used as a covariate to control for baseline between-group imbalances. Following this, standardized thresholds for small, moderate, and large changes (0.2, 0.6, and 1.2, respectively) [42] derived from between-subject SDs of the baseline values were used to assess the magnitude of all effects, with magnitude-based inferences subsequently applied. Here, all inferences were categorized as clinical, with the default probabilities for declaring an effect clinically beneficial being <0.5% (most unlikely) for harm and >25% (possibly) for benefit [42].

## Results

### Measures

When compared with the control group, the effect of the exergaming intervention was a likely small beneficial improvement in predicted VO_2_ max. The SD of the individual responses in predicted VO_2_ max to the exergaming intervention was 3.4 (90% CLs ±3.1). All other effects were either most likely trivial (body mass and waist circumference) or unclear (systolic blood pressure and diastolic blood pressure; Table 1).

### Intervention Delivery

Mean session attendance was 67.5% (170/252; range 0%-100%). Of the 14 intervention participants, 9 attended at least 75% of the prescribed exergaming sessions. The total number of repetitions performed was 1268 (range 6-136 per participant), and the descriptive training data for the exergaming intervention are presented in Table 2. The magnitude of the within-participant variability in all intensity measures was moderate.

**Figure 5:**
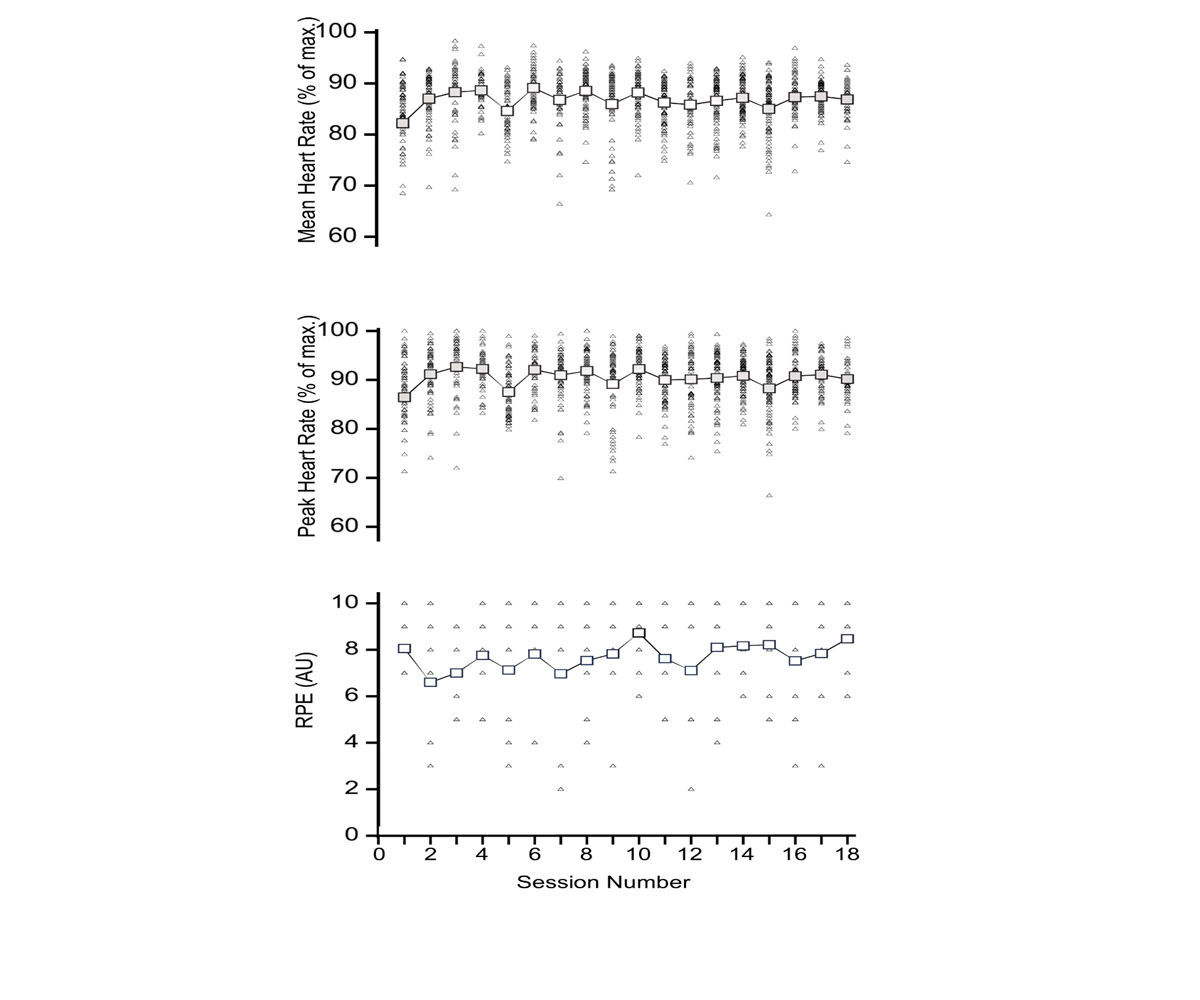
Group mean (large open squares) and individual (small closed triangles) heart rates (session mean and session peak) and ratings of perceived exertion (RPE) across the 6-week exergaming intervention period (session numbers 1-18). AU: arbitrary unit.

Mean exercise intensity data for the 10-s, 20-s, and 30-s repetitions are presented in Table 3. The effect of repetition duration on mean heart rate was substantially higher heart rates during the 30-s repetitions when compared with the 20-s (2.2 percentage points; ±90% CLs 0.5 percentage points) and 10-s repetitions (3.5 percentage points; ±0.5 percentage points). Peak heart rates were also substantially higher for the 30-s repetitions when compared with the 20-s (3.6 percentage points; ±0.6 percentage points) and 10-s repetitions (6.4 percentage points; ±0.6 percentage points). The effect of repetition duration on RPE was most likely trivial. The mean exercise intensity scores per exercise session along with the individual data points to illustrate the variability around the mean score are presented in Figure 5. Across the intervention, the regression slope revealed most likely trivial changes in mean heart rate (0.1 percentage points; ±90% CLs 0.2 percentage points), peak heart rate (0.1 percentage points; ±0.2 percentage points), and RPE (0.1 AU; ±0.1 AU) across the duration of the 6-week HIT intervention.

**Table 1:** Outcome measures at baseline along with the analysis of covariance adjusted change scores and the between-group comparisons of the change scores.

Outcome measures	Intervention group (n=13)		Control group (n=8)		Group comparison^a^
	Baseline values, mean (SD)	Change score, mean (SD)	Baseline values, mean (SD)	Change score, mean (SD)	Difference between groups, % mean; ±90% CL^b^
Body mass (kg)	87 (22)	−1.1 (2.0)	88 (20)	−0.5 (1.6)	−0.5; ±1.4
Waist circumference	97 (15)	−0.6 (1.6)	100 (14)	−0.3 (1.6)	−0.3; ±1.3
Predicted VO_2_ max^c^ (mL/kg/min)	43.7 (8.8)	3.2 (4.1)	39.5 (8.5)	0.2 (2.4)	3.0; ±2.6
Systolic blood pressure	130 (9)	−5.9 (5.5)	134 (12)	−2.7 (6.7)	−3.2; ±5.2
Diastolic blood pressure	80 (10)	−5.1 (5.7)	86 (9)	−5.1 (7.1)	−0.1; ±5.8

^a^High-intensity training (HIT) control.

^b^CL: confidence limit.

^c^VO_2_ max: maximal oxygen consumption.

**Table 2:** Exercise intensity data for the high-intensity training (HIT) intervention.

Intensity measure	Mean (SD)	Within-subject variability; ±90% CL^a^
Mean heart rate (%)	86.3 (5.4)	4.7; ±0.2
Peak heart rate (%)	89.9 (6.1)	5.7; ±0.2
Session RPE^b^ (AU^c^)	7.5 (2.2)	1.6; ±0.1

^a^CL: confidence limit.

^b^RPE: rating of perceived exertion.

^c^AU: arbitrary unit.

**Table 3:** Exercise intensity data for the high-intensity training (HIT) repetition duration.

Intensity measure	10-s repetitions	20-s repetitions	30-s repetitions
Mean heart rate (%)	84.7 ± 5.3	85.9 ± 6.6	88.2 ± 3.5
Peak heart rate (%)	86.8 ± 4.9	89.5 ± 7.7	93.2 ± 3.4
Session RPE^a^ (AU^b^)	7.4 ± 2.2	7.5 ± 2.4	7.6 ± 2.0

^a^RPE: rating of perceived exertion.

^b^AU: arbitrary unit.

### Qualitative Findings

Participants who engaged in interviews ranged between 25 and 50 years and attended between 60% and 100% of the exercise sessions. Data revealed that participants found the exergame to be challenging but rewarding, as illustrated in the following quotes:

At the start you think this is too hard but as you go on and it does get easier, I say it gets easier but you extend the time don’t you so you’re doing more. Half way through the second week I started to appreciate that, what you’re doing is making it harder to keep testing me, do you know what I mean? And you can feel it, you can feel it that you are getting better at the game. I think it still knacks you out [exhausts you] because you start trying harder as it goes on.

Participant 1, 50 years, social club worker

At the beginning it was killing me [exhausting me], but as I carried it on it was still killing me and then it started to get like, better, my heart wasn’t working as hard and I was getting more fit to the point where I coped well, to the point where I was actually still working my hardest at the level I wanted to.

Participant 4, 25 years, hospital worker

Participants found that the competitive aspects resulted in a changing game scenario between players and rounds for each training session, which facilitated motivation to attend and engage, as illustrated in the following quotes:

There was always something else that kept you interested, kept you wanting to do more. If you go for a run, its more like “I’m just running,” that’s it. The fact that you were fighting someone, a different opponent—you had to change, you either didn’t have to work as hard [or you should be] or you had to work harder than you could.

Participant 4, 45 years, hotel worker

I just thought it was a fun way to exercise. If it wasn’t competition, it would be hard to be doing that 2-3 times a week to train at that intensity. Like you would go the odd time training that intense but if it’s against someone you’re more likely to put 100% in. You know, if my heart rate had have got that high just doing it by myself, maybe once or twice but not on a consistent thing like that, in a competition against someone else it does push you to train harder, it did me, it did push me to train harder.

Participant 2, 25 years, unemployed

The variance in the allocated workload was a motivating factor but highlighted that repeated 30-s bursts may have discouraged one participant from attending exercise sessions, as illustrated in the following quotes:

It was probably just right, just right really and I liked how it varied 10, 20, 30—I didn’t want to do 30 seconds all the time.

Participant 3, 34 years, community worker

I always felt as though the 10 seconds were mad [hard] to be honest, because no sooner than you were like finished, then you had to wait about 30 seconds before a 20 second one started or something like that. I loved the 30 second ones. I thought that was great, that was just enough, like 30 seconds in that one round and that was just more than enough sort of thing, you know?

Participant 4, 45 years, hotel worker

Finally, some gameplay elements such as heart rate integration and visual feedback of a successful punch were encouraging for participants, as illustrated in the following quote:

All I knew that if I see the ring go round the head, the further round it is, if I hit him the more points I’d get, so I knew that and I knew every time it flashed I’d caught him. I knew my heart rate was there and I knew the timer was ticking down at the bottom. But the movements and everything else were excellent.

Participant 1, 50 years, social club worker

## Discussion

### Principal Findings

This study developed a novel HIT intervention using exergaming. Obtaining information about whether the game was used as intended by the target population is a fundamental stage in the development cycle. We evaluated its use in men recruited from regions of socioeconomic deprivation, a population predisposed to low life expectancy. The training data is indicative of a consistent and progressive dose of HIT (>85% of maximal heart rate) over the duration of the 6-week intervention. Given that our protocol for measuring training intensity is conservative [38], we are confident that the game was used by the participants as intended. Furthermore, we also found clear beneficial effects on predicted VO_2_ max, and these findings together demonstrate the potential usefulness of exergaming for the future delivery of HIT.

Despite the respective popularities of both HIT and video gaming, there have been no previous attempts to combine these concepts in the form of an exercise intervention. As such, there are no studies against which to directly compare our training data, although the intensity levels are clearly greater than those measured for commercially available exergames [18]. In fact, our training data are more comparable to those from a recent high-intensity exercise programs delivered via traditional means and described as a high-quality dose of HIT [38]. For example, our repetition peak heart rates of 89% of maximal when averaged over the duration of the 6-week intervention were only two percentage points below their levels. Although we cannot pinpoint the specific mechanisms underpinning this success, we suggest the feedback and point-scoring made a strong contribution. Specifically, by matching point-scoring to current training intensity and enabling users to self-regulate and maximize their intensity, it was possible to deliver a consistent dose of HIT. Furthermore, given that these levels were based on their own individual maximal levels (ie, relative to maximal heart rate), the participants were also aware that these were attainable (eg, [44]). Accordingly, after the first session during which users became familiar with the game concepts, intensity levels remained consistent throughout the 6-week intervention, indicative of a high-quality dose of HIT.

When our outcome data are compared with those from other HIT programs, however, our improvements in fitness were relatively modest. For example, the effect on predicted VO_2_ max was only half the pooled effect for untrained males of 6.2% [5]. This relatively smaller improvement could be because of a range of factors (eg, age), although given that we analyzed the outcome data on an intention-to-treat (ITT) basis, nonattendance was the most probable cause. Specifically, our attendance of 68% is much lower than is typical for lab-based studies and more similar to other community-based trials involving HIT. For example, our attendance data was similar to other boxing themed interventions (79%, [45]), mixed-gender HIT programs (75% for maximal volitional intensity training; [46]), obese males (57%, [47]), and community-based HIT interventions using adult populations (58%-77%, [48]). Thus, although there were some positive findings related to this HIT intervention (eg, ease of recruitment in the target population and training at the intended dose), some of the usual problems associated with conducting exercise interventions in the community remained. Despite favorable changes in VO_2_ max, there were no clear beneficial effects for body mass, waist circumference, and blood pressure when compared with our control group. We suspect that our ITT analysis and shorter intervention period (<12 weeks) influenced these outcome measures when compared with other HIT studies [35,49].

In summary, we have established that HIT can be delivered via exergaming but that the usual problems of attendance remain. Clearly, finding strategies to improve attendance will be important in future development cycles. One of the appealing features of HIT among our participants was the time-efficient nature of HIT. However, the delivery of our exergaming sessions in many cases required travel to and from the venue on a thrice-weekly basis. This could add hours onto a relatively brief period of HIT, and for some, this was a major disincentive to attend. On a more positive note, however, online gaming is now a major part of the gaming industry, and through internet protocols (IPs) and greater bandwidth, these exergaming sessions could be delivered over the internet. Further work into the acceptability of delivering exercise in this manner may need to be undertaken, but the benefits of doing exercise at work or at home would most probably appeal to some of our participants. Furthermore, by using these technologies effectively, it may be possible to exploit other opportunities to further motivate, such as social media to improve competitiveness and camaraderie [50].

### Limitations

There were several limitations to this study that need to be overcome in a larger trial. First, our sample only included white Europeans and South Asian males recruited from regions of socioeconomic deprivation. The acceptability of this intervention across different ethnicities and other socioeconomic groups is therefore not known. Second, we administered a submaximal fitness test despite it being known that such tests are prone to errors [51]. Third, minigames such as this usually have a short lifespan [52,53], and it is not known how much longer the exergame could maintain interest beyond this 6-week intervention. Future studies are needed to explore the acceptability of a maximal VO_2_ max test within this population. Fourth, participant blinding was not possible in this study, and we therefore cannot rule out cluster contamination and participant allocation resentment. Fifth, we did not perform an economic evaluation of this intervention. Whether these benefits, given the costs of treating symptoms associated with lack of exercise, set against the costs of developing and delivering the intervention, requires formal evaluation in future research.

### Conclusions

Exergaming can be configured to deliver a sustained and consistent dose of high-intensity exercise, resulting in improved fitness in a group of males living in regions of socioeconomic status over a 6-week program. Furthermore, class-salient interventions delivered to specific populations have the potential to facilitate adherence and attendance. Exergaming is inherently scalable and has good reach into our population, although future strategies using IPs could improve the real-world effectiveness.

## Abbreviations

AU: arbitrary unit
CL: confidence limit
HIT: high-intensity training
HRmax: maximal heart rate
IP: internet protocol
MPID: minimal practically important difference
RPE: rating of perceived exertion
VO_2_ max: maximal oxygen consumption

## Appendix

Multimedia Appendix 1 CONSORT‐EHEALTH checklist (V 1.6.1).
