# Supplementary material for: Development of an Exergame to Deliver a Sustained Dose of High-Intensity Training: Formative Pilot Randomized Trial
Source: JMIR Serious Games. 2018 Mar 27;6(1):e4. doi: 10.2196/games.7758 (PMC5893890; doi:10.2196/games.7758)
Supplement: Multimedia Appendix 1 [file games_v6i1e4_app1.pdf]

| CONSORT-EHEALTH Checklist V1.6.2 Report | Manuscript Number | 7758 |
|---|---|---|
| **(based on CONSORT-EHEALTH V1.6), available at [http://tinyurl.com/consort-ehealth-v1-6].** | | |
| 7758 | | |
| **Date completed** | | |
| 3/4/2018 16:58:13 | | |
| **by** | | |
| Thomas McBain | | |
| | | |
| | | |
| TITLE | | |
| **1a-i) Identify the mode of delivery in the title** | | |
| | | |
| **1a-ii) Non-web-based components or important co-interventions in title** | | |
| "Exergame" | | |
| **1a-iii) Primary condition or target group in the title** | | |
| No | | |
| ABSTRACT | | |
| **1b-i) Key features/functionalities/components of the intervention and comparator in the METHODS section of the ABSTRACT** | | |
| | | |
| **1b-ii) Level of human involvement in the METHODS section of the ABSTRACT** | | |
| Yes. "The intervention was a 6-week training program consisting of three sessions of exergaming per week. The sessions involved a structured warm-up, then brief intermittent repetitions in the form of boxing rounds (10 s, 20 s, and 30 s) against their peers with a work/rest ratio of 0.25." | | |
| **1b-iii) Open vs. closed, web-based (self-assessment) vs. face-to-face assessments in the METHODS section of the ABSTRACT** | | |
| No. There is reference to warm-ups and HIT repetitions performed against their peers within the abstract and further reference to the two locations that participants were recruited from for delivery of the sessions within the manuscript. | | |
| **1b-iv) RESULTS section in abstract must contain use data** | | |
| No. The abstract focuses on the delivery and effect of HIT on selected health outcomes. Initially, the abstract outlined the population but this was later omitted. | | |
| **1b-v) CONCLUSIONS/DISCUSSION in abstract for negative trials** | | |
| Yes. Methods; "participants were allocated to intervention (n=14) or control groups (n=10) by third-party minimization". "Retention to the intervention was 87.5% (21/24). Over the duration of the intervention, session attendance was 67.5% (170/252)". Additional data is supplied regarding the primary outcome measures and physiological response to the intervention. | | |
| INTRODUCTION | | |
| **2a-i) Problem and the type of system/solution** | | |
| | | |
| **2a-ii) Scientific background, rationale: What is known about the (type of) system** | | |

| | | |
|---|---|---|
| Yes. "The latest government audit of university-based research in the United Kingdom has highlighted the need for more sport and exercise research to target health inequalities that persist in society [1]. There are strong inverse relationships between life expectancy, cardiorespiratory fitness, and socioeconomic status [2-4]. As these trends are exacerbated in males, improving<br>fitness in men living in regions of socioeconomic deprivation could be important for addressing health inequalities. High-intensity training (HIT) is a time-efficient way to improve fitness over a short duration [5], and a growing body of evidence<br>advocates this form of exercise for public health interventions [6]. HIT is known to stimulate a combination of central and peripheral adaptations promoting an enhanced availability, extraction, and utilization of oxygen [7,8]. Although these<br>findings are encouraging, these previous studies have tended to prescribe exercise with a focus on cycling and running [9-12]. Some argue that the psychologically aversive nature of high-intensity exercise means that this training will not be adopted or maintained by many people [13]. Thus, although the physiological benefits of HIT are unequivocal, there is ongoing debate about its relevance across populations, particularly for those less-motivated individuals. Finding ways to improve its acceptability could be the key to improve the wider acceptance of HIT.". "A major challenge with improving HIT and exergaming is to translate positive laboratory-based findings into interventions<br>that can directly affect those individuals who need it most and can also be administered on a larger scale [16,17]. Whether or not the concept of serious exergaming can contribute in this regard requires, first, that a game is tailored toward reducing the aversive protocols associated with HIT and second, that the<br>intervention is capable of improving fitness in our target population". "A major challenge with improving HIT and exergaming is to translate positive laboratory-based findings into interventions that can directly affect those individuals who need it most and can also be administered on a larger scale [16,17]. Whether or<br>not the concept of serious exergaming can contribute in this regard requires, first, that a game is tailored toward reducing the aversive protocols associated with HIT and second, that the intervention is capable of improving fitness in our target population". | | |
| METHODS | | |
| **3a) CONSORT: Description of trial design (such as parallel, factorial) including allocation ratio** | | |
| | | |
| **3b) CONSORT: Important changes to methods after trial commencement (such as eligibility criteria), with reasons** | | |
| Yes. "our first aim was to describe the development of a serious exergame for this purpose. Our second aim was to quantify its real-world fidelity and potential to improve fitness and health outcomes in males recruited in regions of socioeconomic deprivation over a 6-week intervention." | | |
| **3b-i) Bug fixes, Downtimes, Content Changes** | | |
| | | |
| **4a) CONSORT: Eligibility criteria for participants** | | |
| Yes. "We conducted a 6-week exploratory controlled trial". "A third-party minimization process using baseline measures of age, waist circumference, and predicted maximum oxygen consumption (VO2 max) was used to remove bias in group allocation." | | |
| **4a-i) Computer / Internet literacy** | | |
| | | |
| **4a-ii) Open vs. closed, web-based vs. face-to-face assessments:** | | |
| N/A | | |
| **4a-iii) Information giving during recruitment** | | |
| Yes. "Men are commonly referred to as a hard-to-reach population to engage in health promotion activities [27], where a targeted recruitment approach at locations predominantly attended by men may facilitate uptake of participants. Therefore, to maximize recruitment within our intended population, relevant gatekeepers were approached at institutions positioned within regions of social deprivation. Thus, two settings used for recruitment and the trials were a social club and mosque, both situated within deprived regions of Middlesbrough, United Kingdom (TS1 and TS4). Recruitment in the social club relied heavily on the gatekeeper (club secretary), whereas uptake of South Asian men from a local mosque was more straightforward; all 9 mosque participants were recruited from a single demonstration (and we had similar experiences in previous iterations)." | | |
| **4b) CONSORT: Settings and locations where the data were collected** | | |
| N/A. There were no changes to the trial after commencement | | |
| **4b-i) Report if outcomes were (self-)assessed through online questionnaires** | | |
| | | |
| **4b-ii) Report how institutional affiliations are displayed** | | |
| N/A. Health outcomes were assessed in-person by a member of the research team [TM]. | | |
| **5) CONSORT: Describe the interventions for each group with sufficient details to allow replication, including how and when they were actually administered** | | |

| | | |
|---|---|---|
| **5-i) Mention names, credential, affiliations of the developers, sponsors, and owners** | | |
| | | |
| **5-ii) Describe the history/development process** | | |
| N/A | | |
| **5-iii) Revisions and updating** | | |
| Yes. The paper has sub-section titled "Development of the Game and Intervention" where in-depth details are provided regarding focus groups and software/hardware development for the trial. | | |
| **5-iv) Quality assurance methods** | | |
| N/A. No revisions or updates were made to the trail, software and hardware after commencement. | | |
| **5-v) Ensure replicability by publishing the source code, and/or providing screenshots/screen-capture video, and/or providing flowcharts of the algorithms used** | | |
| N/A | | |
| **5-vi) Digital preservation** | | |
| Yes. A flowchart for the machine state variables used for delivering a HIT dose are provided in a figure within the text. Furthermore, details about the development of the software are provided in the manuscript. | | |
| **5-vii) Access** | | |
| N/A | | |
| **5-viii) Mode of delivery, features/functionalities/components of the intervention and comparator, and the theoretical framework** | | |
| Yes. Allocation to groups was randomized and is highlighted in figure 4 (participant flow). "two settings used for recruitment and the trials were a social club and mosque". Participants allocated to the intervention group were able to play the exergame during the 6-week period. | | |
| **5-ix) Describe use parameters** | | |
| Yes. A sub-section of the method section is titled "Intervention Delivery"; Evidence recommends a minimum duration of 12 weeks for a HIT protocol to promote favorable changes in blood pressure and anthropometric measurements of obesity [35]. However, a 6-week intervention was selected, as a minimum of 13 sessions (0.16 work/rest ratio) is sufficient to elicit moderate improvements in VO2 max in sedentary individuals [5]. Additionally, there is still ambiguity regarding the optimal work-to-rest ratio when designing HIT interventions, particularly in populations with varied age, baseline fitness, and training experience [36]. Therefore, longer duration HIT models (1-4 min) were deemed unsuitable for our target population [37]. Furthermore, minigames (such as the current exergame) have short life spans, where adherence to a longer intervention (eg, 12 weeks) may diminish over time and influence health outcomes. This was evident from a 12-week pilot study (unpublished data) using an exergame in the same population that saw attendance drop from 53% during week 2 to 16% during week 12. At the beginning of the exergaming session, participants were required to complete a 6-min structured warm-up consisting of a series of exercises on a 210 mm step until both participants reached >70% HRmax. Session workloads with volumetric progression were set automatically once the user’s identifying information was entered. The session workloads were 120s-, 150-s, and 180-s of work during weeks 1 and 2, weeks 3 and 4, and weeks 5 and 6, respectively. To avoid staleness, the repetition lengths (10, 20, or 30-s) were randomly selected at the beginning of each round. We set the work-to-rest ratio at 1:4, and thus, the respective repetitions were followed by 40, 80, or 120-s of active recovery. Each exergaming session took approximately 30 to 40 min to complete, including equipment set-up, warm-up with additional enjoyment, and task immersion questionnaires upon completion of the HIT bouts (not reported here). Heart rate responses were taken within repetitions and therefore, did not include any of the recovery period. This, therefore, avoided an overestimation of physiological load, which can occur when heart rate continues to rise after exercise cessation [26].". "To explore perceptions of the exergame and the HIT regime, semistructured interviews were conducted with 5 intervention participants following the 6-week training period, which were analyzed semantically." | | |
| **5-x) Clarify the level of human involvement** | | |
| “Participants allocated to the intervention group were invited to attend three sessions of exergaming per week”. "At the beginning of the exergaming session, participants were required to complete a 6-min structured warm-up consisting of a series of exercises on a 210 mm step" | | |
| **5-xi) Report any prompts/reminders used** | | |
| No. Although this is not mentioned in the paper, a member of the research team [TM] was responsible for setting up the equipment and running the HIT sessions. | | |
| **5-xii) Describe any co-interventions (incl. training/support)** | | |
| No. Regular contact was made by a member of the research team [TM] to organize training sessions. | | |
| **6a) CONSORT: Completely defined pre-specified primary and secondary outcome measures, including how and when they were assessed** | | |

| | | |
|---|---|---|
| Yes. "Inclusion criteria were deliberately broad to maximize recruitment, that is, apparently healthy, as defined by ACSM guidelines and in the age range of 18 to 55 years" | | |
| **6a-i) Online questionnaires: describe if they were validated for online use and apply CHERRIES items to describe how the questionnaires were designed/deployed** | | |
| | | |
| **6a-ii) Describe whether and how "use" (including intensity of use/dosage) was defined/measured/monitored** | | |
| N/A | | |
| **6a-iii) Describe whether, how, and when qualitative feedback from participants was obtained** | | |
| Yes. Heart rate was collected during the trails and specified "two wireless chest-worn heart-rate monitors (Polar RS400, Polar Electro Oy, Kempele, Finland).". ""Participants were instructed to perform the repetitions at an intensity ≥85% HRmax"" | | |
| **6b) CONSORT: Any changes to trial outcomes after the trial commenced, with reasons** | | |
| Yes. "two settings used for recruitment and the trials were a social club and mosque, both situated within deprived regions of Middlesbrough, United Kingdom (TS1 and TS4)" | | |
| **7a) CONSORT: How sample size was determined** | | |
| **7a-i) Describe whether and how expected attrition was taken into account when calculating the sample size** | | |
| | | |
| **7b) CONSORT: When applicable, explanation of any interim analyses and stopping guidelines** | | |
| Yes. "All measures were assessed pre and post intervention. Blood pressure was collected on the left arm positioned at heart height with the subjects in a seated position by an automatic upper arm blood pressure monitor (Omron MX13). Measures were made at least three times at 3-min intervals, where an average of the two lowest measures was used for analysis [31]. Waist circumference was measured using the World Health Organization guidelines [32]. Predicted VO2 max was obtained by performing a submaximal 8-min ramped step test [33]. Heart rate response (Polar T34; PolarElectro OY, Kempele, Finland) and simultaneous breath-by-breath expired gas were collected<br>using a portable indirect calorimeter (Cosmed K4 b2; Rome, Italy), calibrated per the manufacturer's guidelines. Individual HRmax was estimated [34] and plotted against VO2 data for the determination of predicted VO2 max.". "Body mass index [29] and waist circumference [30] cut-points were adjusted for ethnicity." | | |
| **8a) CONSORT: Method used to generate the random allocation sequence** | | |
| N/A | | |
| **8b) CONSORT: Type of randomisation; details of any restriction (such as blocking and block size)** | | |
| N/A | | |
| **9) CONSORT: Mechanism used to implement the random allocation sequence (such as sequentially numbered containers), describing any steps taken to conceal the sequence until interventions were assigned** | | |
| Yes. "A third-party minimization process using baseline measures of age, waist circumference, and predicted maximum oxygen consumption (VO2 max) was used to remove bias in group allocation." | | |
| **10) CONSORT: Who generated the random allocation sequence, who enrolled participants, and who assigned participants to interventions** | | |
| Yes. "A third-party minimization process using baseline measures of age, waist circumference, and predicted maximum oxygen consumption (VO2 max) was used to remove bias in group allocation." | | |
| **11a) CONSORT: Blinding - If done, who was blinded after assignment to interventions (for example, participants, care providers, those assessing outcomes) and how** | | |
| **11a-i) Specify who was blinded, and who wasn't** | | |
| | | |
| **11a-ii) Discuss e.g., whether participants knew which intervention was the "intervention of interest" and which one was the "comparator"** | | |
| N/A | | |
| **11b) CONSORT: If relevant, description of the similarity of interventions** | | |
| Yes. "A third-party minimization process using baseline measures of age, waist circumference, and predicted maximum oxygen consumption (VO2 max) was used to remove bias in group allocation." | | |
| **12a) CONSORT: Statistical methods used to compare groups for primary and secondary outcomes** | | |
| No | | |
| **12a-i) Imputation techniques to deal with attrition / missing values** | | |
| | | |

| | | |
|---|---|---|
| **12b) CONSORT: Methods for additional analyses, such as subgroup analyses and adjusted analyses** | | |
| N/A | | |
| RESULTS | | |
| **13a) CONSORT:  For each group, the numbers of participants who were randomly assigned, received intended treatment, and were analysed for the primary outcome** | | |
| Yes | | |
| **13b) CONSORT:  For each group, losses and exclusions after randomisation, together with reasons** | | |
| N/A | | |
| **13b-i) Attrition diagram** | | |
| | | |
| **14a) CONSORT: Dates defining the periods of recruitment and follow-up** | | |
| Yes. This information is presented in Figure 4. | | |
| **14a-i) Indicate if critical “secular events” fell into the study period** | | |
| | | |
| **14b) CONSORT: Why the trial ended or was stopped (early)** | | |
| Yes. This information is presented in Figure 4. | | |
| **15) CONSORT: A table showing baseline demographic and clinical characteristics for each group** | | |
| Yes. "The intervention took place from November 2014 to December 2014 (social club) and March 2015 to April 2015 (mosque)." | | |
| **15-i) Report demographics associated with digital divide issues** | | |
| | | |
| **16a) CONSORT: For each group, number of participants (denominator) included in each analysis and whether the analysis was by original assigned groups** | | |
| **16-i) Report multiple “denominators” and provide definitions** | | |
| | | |
| **16-ii) Primary analysis should be intent-to-treat** | | |
| Yes. Information displayed in Table 1. | | |
| **17a) CONSORT: For each primary and secondary outcome, results for each group, and the estimated effect size and its precision (such as 95% confidence interval)** | | |
| N/A | | |
| **17a-i) Presentation of process outcomes such as metrics of use and intensity of use** | | |
| | | |
| **17b) CONSORT: For binary outcomes, presentation of both absolute and relative effect sizes is recommended** | | |
| Yes. Table 1 depicts this information | | |
| **18) CONSORT: Results of any other analyses performed, including subgroup analyses and adjusted analyses, distinguishing pre-specified from exploratory** | | |
| Yes. Tables 1, 2 and 3 display this information | | |
| **18-i) Subgroup analysis of comparing only users** | | |
| | | |
| **19) CONSORT: All important harms or unintended effects in each group** | | |
| N/A | | |
| **19-i) Include privacy breaches, technical problems** | | |
| | | |
| **19-ii) Include qualitative feedback from participants or observations from staff/researchers** | | |
| No. There were no negative consequences experienced during the intervention. | | |
| DISCUSSION | | |
| **20) CONSORT: Trial limitations, addressing sources of potential bias, imprecision, multiplicity of analyses** | | |

| | | |
|---|---|---|
| **20-i) Typical limitations in ehealth trials** | | |
| | | |
| **21) CONSORT: Generalisability (external validity, applicability) of the trial findings** | | |
| **21-i) Generalizability to other populations** | | |
| | | |
| **21-ii) Discuss if there were elements in the RCT that would be different in a routine application setting** | | |
| Yes. " First, our sample only included white Europeans and South Asian males recruited from regions of socioeconomic deprivation. The acceptability of this intervention across different ethnicities and other socioeconomic groups is<br>therefore not known" | | |
| **22) CONSORT: Interpretation consistent with results, balancing benefits and harms, and considering other relevant evidence** | | |
| **22-i) Restate study questions and summarize the answers suggested by the data, starting with primary outcomes and process outcomes (use)** | | |
| | | |
| **22-ii) Highlight unanswered new questions, suggest future research** | | |
| Yes. "This study developed a novel HIT intervention using exergaming. Obtaining information about whether the game was used as intended by the target population is a fundamental stage in the development cycle. We evaluated its use in men recruited from regions of socioeconomic deprivation, a population predisposed to low life expectancy. The training data is indicative of a consistent and progressive dose of HIT (>85% of maximal heart rate) over the duration of the 6-week intervention. Given that our protocol for measuring training intensity is conservative [38], we are confident that the game was used by the participants as intended. Furthermore, we also found clear beneficial effects on predicted VO2 max, and these findings together demonstrate the potential usefulness of<br>exergaming for the future delivery of HIT."<br>"Despite the respective popularities of both HIT and video gaming, there have been no previous attempts to combine these concepts in the form of an exercise intervention. As such, there are no studies against which to directly compare our training data, although the intensity levels are clearly greater than those measured for commercially available exergames [18]. In fact, our training data are more comparable to those from a recent high-intensity exercise programs delivered via traditional means and described as a high-quality dose of HIT [38]. For example, our repetition peak heart rates of 89% of maximal when averaged over the duration of the 6-week intervention were only two percentage points below their levels. Although we cannot pinpoint the specific mechanisms underpinning this success, we suggest the feedback and point-scoring made a strong contribution. Specifically, by matching point-scoring to current training intensity and enabling users to self-regulate and maximize their intensity, it was possible to deliver a consistent dose of HIT. Furthermore, given that these levels were based on their own individual maximal levels (ie, relative to maximal heart rate), the participants were also aware that these were attainable (eg, [44]). Accordingly, after the first session during which users became familiar with the game concepts, intensity levels remained consistent throughout the 6-week intervention, indicative of a high-quality dose of HIT."<br>"When our outcome data are compared with those from other HIT programs, however, our improvements in fitness were relatively modest. For example, the effect on predicted VO2 max was only half the pooled effect for untrained males of 6.2% [5]. This relatively smaller improvement could be because of a range of factors (eg, age), although given that we analyzed the outcome data on an intention-to-treat (ITT) basis, nonattendance was the most probable cause. Specifically, our attendance of 68% is much lower than is typical for lab-based studies and more similar to other community-based trials involving HIT. For example, our attendance data was similar to other boxing themed interventions (79%, [45]), mixed-gender HIT programs (75% for maximal volitional intensity training; [46]), obese males (57%, [47]), and community-based HIT interventions<br>using adult populations (58%-77%, [48]). Thus, although there were some positive findings related to this HIT intervention (eg, ease of recruitment in the target population and training at the intended dose), some of the usual problems associated with conducting exercise interventions in the community remained.<br>Despite favorable changes in VO2 max, there were no clear beneficial effects for body mass, waist circumference, and blood pressure when compared with our control group. We suspect that our ITT analysis and shorter intervention period (<12 weeks) influenced these outcome measures when compared with other HIT studies [35,49]. | | |
| Other information | | |
| **23) CONSORT: Registration number and name of trial registry** | | |
| N/A | | |
| **24) CONSORT: Where the full trial protocol can be accessed, if available** | | |
| N/A | | |
| **25) CONSORT: Sources of funding and other support (such as supply of drugs), role of funders** | | |
| No | | |
| **X26-i) Comment on ethics committee approval** | | |

| | | |
|---|---|---|
| | | |
| **x26-ii) Outline informed consent procedures** | | |
| Yes. "The study was approved by the ethics committee of Teesside University, United Kingdom, and written informed consent was obtained from all participants." | | |
| **X26-iii) Safety and security procedures** | | |
| Yes. "written informed consent was obtained from all participants." | | |
| **X27-i) State the relation of the study team towards the system being evaluated** | | |
